# Relevance of In Vitro Metabolism Models to PET Radiotracer Development: Prediction of In Vivo Clearance in Rats from Microsomal Stability Data

**DOI:** 10.3390/ph12020057

**Published:** 2019-04-14

**Authors:** Daniela Schneider, Angela Oskamp, Marcus Holschbach, Bernd Neumaier, Andreas Bauer, Dirk Bier

**Affiliations:** 1 Institute of Neuroscience and Medicine—Molecular Organization of the Brain (INM-2), Forschungszentrum Jülich GmbH, 52428 Jülich, Germany; a.oskamp@fz-juelich.de (A.O.); an.bauer@fz-juelich.de (A.B.); 2 Institute of Neuroscience and Medicine—Nuclear Chemistry (INM-5), Forschungszentrum Jülich GmbH, 52428 Jülich, Germany; m.holschbach@fz-juelich.de (M.H.); b.neumaier@fz-juelich.de (B.N.); d.bier@fz-juelich.de (D.B.); 3 Neurological Department, Medical Faculty, Heinrich-Heine-University, Universitätsstraße 1, 40225 Düsseldorf, Germany

**Keywords:** radiotracer, clearance, in vitro-in vivo extrapolation, A_1_ adenosine receptor, [^18^F]CPFPX, PET imaging

## Abstract

The prediction of in vivo clearance from in vitro metabolism models such as liver microsomes is an established procedure in drug discovery. The potentials and limitations of this approach have been extensively evaluated in the pharmaceutical sector; however, this is not the case for the field of positron emission tomography (PET) radiotracer development. The application of PET radiotracers and classical drugs differs greatly with regard to the amount of substance administered. In typical PET imaging sessions, subnanomolar quantities of the radiotracer are injected, resulting in body concentrations that cannot be readily simulated in analytical assays. This raises concerns regarding the predictability of radiotracer clearance from in vitro data. We assessed the accuracy of clearance prediction for three prototypical PET radiotracers developed for imaging the A_1_ adenosine receptor (A_1_AR). Using the half-life (t_1/2_) approach and physiologically based scaling, in vivo clearance in the rat model was predicted from microsomal stability data. Actual clearance could be accurately predicted with an average fold error (AFE) of 0.78 and a root mean square error (RMSE) of 1.6. The observed slight underprediction (1.3-fold) is in accordance with the prediction accuracy reported for classical drugs. This result indicates that the prediction of radiotracer clearance is possible despite concentration differences of more than three orders of magnitude between in vitro and in vivo conditions. Consequently, in vitro metabolism models represent a valuable tool for PET radiotracer development.

## 1. Introduction

The application of PET as a tool for molecular neuroimaging is limited by the availability of suitable radiotracers. In radiotracer development, the in vivo performance of a novel compound is determined by numerous physicochemical and pharmacological factors, of which metabolism represents a particularly important one [1]. The metabolic lability of a candidate radiotracer may lead to a rapid decrease of radiotracer plasma concentration, resulting in insufficient brain exposure. This is particularly problematic if longer scan durations are required to properly image the molecular target. Additionally, excessive radiotracer metabolism increases the risk that brain-penetrant radiolabeled metabolites are generated in sufficient amounts to compromise the PET signal. However, metabolic degradation also supports the fast clearance of radioactivity from the blood pool which, on the one hand, improves the target-to-background ratio obtainable during the PET scan and thus the image contrast, and, on the other hand, allows for shorter scan duration [2,3]. These aspects illustrate the importance of a precise adjustment of the metabolic properties of lead compounds during the radiotracer development process to produce promising imaging agents for in vivo application. Various in vitro techniques are available to evaluate the metabolic stability of novel compounds during the preclinical stage. The potential and limitations of these methods have been extensively evaluated in the field of drug discovery and development [4,5,6,7,8]; however, with regard to the development of radiotracers, studies on the physiological relevance of in vitro metabolism models are rare. The in vivo application of PET radiotracers differs greatly from the application of classical drugs, especially in terms of the amount of substance administered. In a typical PET study, the average body concentration of a radiotracer is in the subnanomolar range. Detection of such low concentrations is usually not feasible with the classical analytical techniques employed in metabolic stability assays, especially if structure determination of metabolites is required in addition. Consequently, in vitro radiotracer metabolism studies typically involve substrate concentrations that do not reflect the in vivo scenario, which raises questions about the physiological relevance and predictive power of the generated data that go beyond the fundamental concerns on in vitro system performance arising from classical drug evaluation studies.

**Figure 1:**
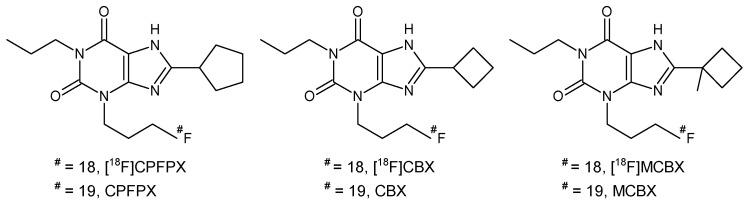
Structures of 8-cyclopentyl-3-(3-[^18^F]fluoropropyl)-1-propylxanthine ([^18^F]CPFPX), 8-cyclobutyl-3-(3-[^18^F]fluoropropyl)-1-propylxanthine ([^18^F]CBX), 3-(3-[^18^F]fluoropropyl)-8-(1-methylcyclobutyl)-1-propylxanthine ([^18^F]MCBX) and their nonradioactive counterparts.

In this study, we compared preclinical in vitro and in vivo clearance data of three xanthine-based radioligands for the A_1_AR. Structural analogs of the methylxanthine caffeine are an important class of A_1_AR antagonists [9] which, when labeled with a positron-emitting radionuclide such as ^11^C or ^18^F, enable the in vivo visualization of the A_1_AR with PET. To date, the ^18^F-labeled compound 8-cyclopentyl-3-(3-[^18^F]fluoropropyl)-1-propylxanthine ([^18^F]CPFPX, Figure 1) [10,11], which was the first radiolabeled A_1_AR ligand used in human PET studies [12], is still considered the gold standard for in vivo imaging of the A_1_AR. Numerous human and animal imaging studies have been successfully conducted using [^18^F]CPFPX [13,14,15,16]; however, since this radiotracer undergoes rapid metabolic degradation [17,18], continuous efforts have been made to develop metabolically stable analogs that may provide higher image quality during PET scans [19]. Recognizing the C8-substituent at the xanthine core as a main target of metabolic enzymes [17], the development process concentrated predominantly on the synthesis of C8-substituted analogs of [^18^F]CPFPX. In the present preclinical study, the predictability of radiotracer in vivo clearance from microsomal stability data was evaluated using [^18^F]CPFPX and two novel cyclobutyl analogs, namely 8-cyclobutyl-3-(3-[^18^F]fluoropropyl)-1-propylxanthine ([^18^F]CBX) and 3-(3-[^18^F]fluoropropyl)-8-(1-methylcyclobutyl)-1-propylxanthine ([^18^F]MCBX) as model compounds (Figure 1).

## 2. Results

### 2.1. Stability in Liver Microsomes

**Figure 2:**
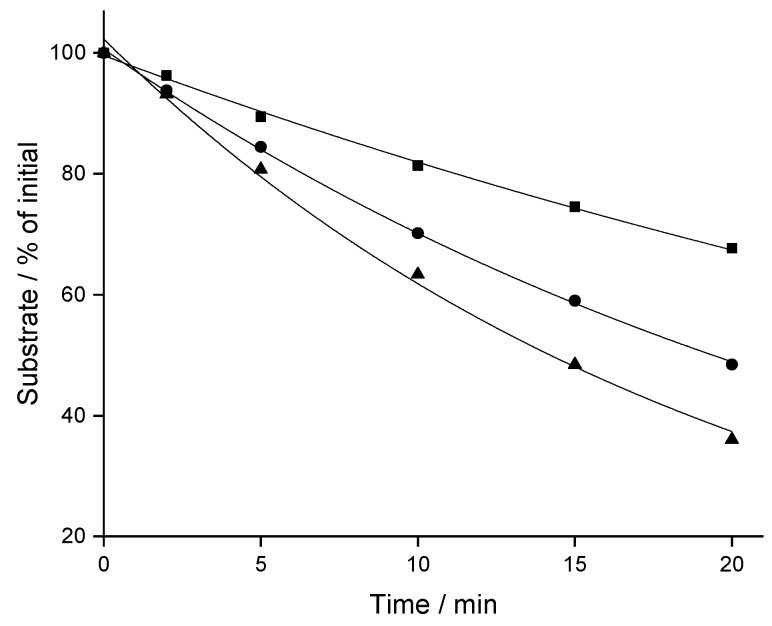
Representative depletion-time profiles of CPFPX (triangles), CBX (squares) and MCBX (circles) in rat liver microsomes. Data were fitted to a monoexponential model (solid lines).

Depletion of CBX, MCBX and CPFPX was evaluated in rat liver microsomes (RLM) at a concentration of 8 µM. Time-courses of substrate disappearance exhibited monoexponential characteristics, as shown in Figure 2 for typical microsomal assays. In vitro t_1/2_ and intrinsic clearance (CL_int_) values derived from the monoexponential fits differed substantially between the three analogous compounds (Table 1), with CBX being the most stable (t_1/2_ = 35.1 min) and CPFPX the least stable analog (t_1/2_ = 14.0 min).

### 2.2. In Vivo Pharmacokinetic (PK) Studies

**Figure 3:**
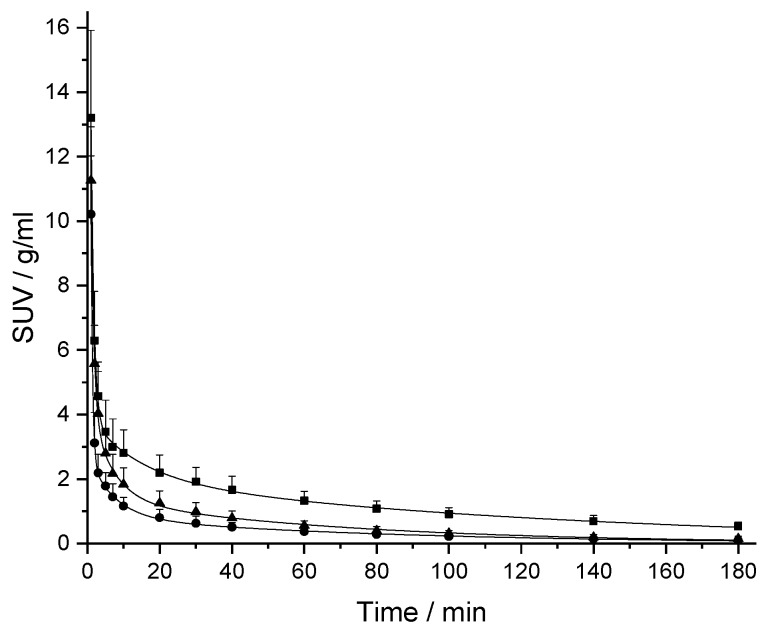
Plasma kinetics of [^18^F]CPFPX (triangles), [^18^F]CBX (squares) and [^18^F]MCBX (circles) in rat after i.v. bolus administration. The standardized uptake value (SUV) was calculated by normalizing the plasma radioactivity concentration to the amount of injected radioactivity and body weight. Solid lines represent the triexponential model fits. Data (mean ± SD) were obtained from eight ([^18^F]CBX, [^18^F]CPFPX) or nine animals ([^18^F]MCBX).

**Figure 4:**
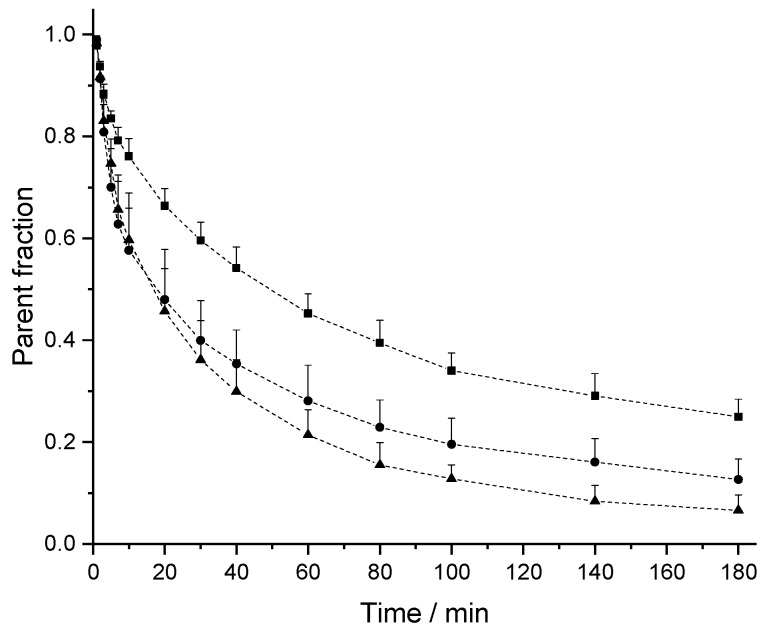
Time course of intact [^18^F]CPFPX (triangles), [^18^F]CBX (squares) and [^18^F]MCBX (circles) in rat plasma. Dashed lines are a guide to the eye. Data (mean ± SD) were obtained from 8 ([^18^F]CBX, [^18^F]CPFPX) or 9 animals ([^18^F]MCBX).

The PK profiles of [^18^F]CBX, [^18^F]MCBX and [^18^F]CPFPX in rat plasma following a single intravenous (i.v.) dose of approximately 0.4 nmol are shown in Figure 3. The examination of the semi-logarithmic standardized uptake values (SUV) versus time plots (not shown) revealed three distinctive kinetic phases associated with the decline of the radiotracer concentration in plasma. Consequently, a triexponential model was chosen for curve fitting. The plasma clearance, volume of distribution (V_d_) and terminal half-life (t_1/2,term_) were estimated from the fitted parameters (Table 2). Volumes of distribution of the compounds ranged from 356–715 mL/kg. Terminal half-lives of [^18^F]CPFPX and [^18^F]MCBX were comparable (approximately 50 min), whereas t_1/2,term_ of [^18^F]CBX was considerably longer (76.5 min). Highest clearance was observed with [^18^F]MCBX (9.10 mL/min/kg), lowest clearance with [^18^F]CBX (3.22 mL/min/kg). Plasma clearance values calculated from individual PK profiles (data not shown) deviated less than 4% from those derived from the mean value curves. The time-courses of parent fraction in plasma are shown in Figure 4. Parent fractions at 1, 2, and 3 min post injection (p.i.) were 98–99%, 91–94% and 81–88%, respectively. At the end of the measurement (180 min p.i.), authentic radiotracer accounted for approximately 7% ([^18^F]CPFPX), 13% ([^18^F]MCBX) and 25% ([^18^F]CBX) of the total plasma radioactivity.

### 2.3. Plasma Protein Binding

**Figure 5:**
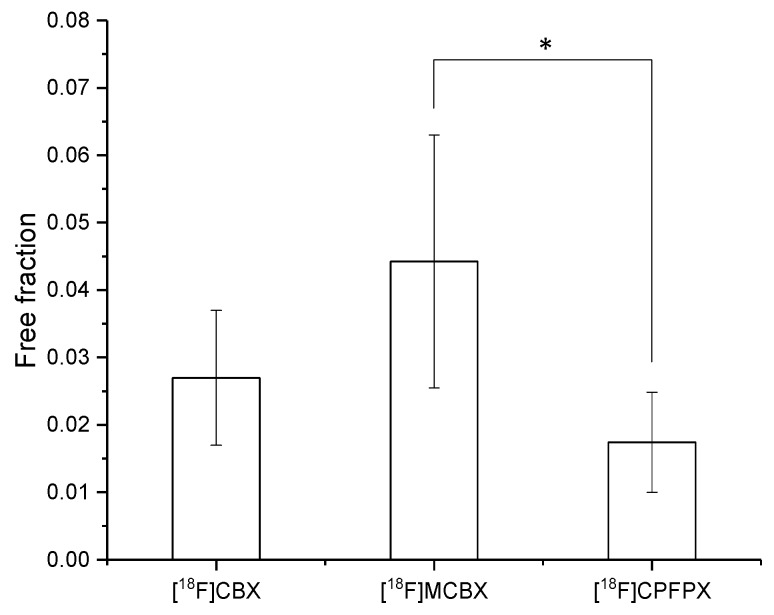
Free fraction of the radiotracers in rat plasma. Data (mean ± SD) were obtained from five ([^18^F]CPFPX) or seven ([^18^F]MCBX, [^18^F]CBX) individual animals. * significantly different (*p* < 0.05, one-way ANOVA with post-hoc Tukey test).

The extent of the binding of [^18^F]CBX, [^18^F]MCBX and [^18^F]CPFPX to rat plasma proteins was determined via ultrafiltration of the spiked plasma samples. The spiked radiotracer concentrations ranged from approximately 0.4–0.6 nM, resembling in vivo concentrations. All three compounds exhibited high plasma protein binding with resulting free fractions of less than 5% (Figure 5).

### 2.4. Prediction of In Vivo Clearance from In Vitro Data

The in vivo predicted plasma clearance (CL_p_) of CBX, MCBX and CPFPX were calculated from microsomal stability data according to Equations (2)–(4). Corrections were applied for microsomal and plasma protein binding. As can be seen from Table 2, the actual in vivo CL_p_ of the three compounds in rat were accurately predicted by calculated CL_p_ values with an average fold error (AFE) of 0.78 (corresponding to an average fold underprediction of 1.3) and a root mean square error (RMSE) of 1.6. All predictions fell within 1-fold of the observed value. Underprediction was largest for CPFPX (fold error of 0.66) and smallest for CBX (fold error of 0.84).

## 3. Discussion

The prediction of in vivo metabolic stability from hepatic cellular and subcellular systems is an integral part of drug discovery. It is widely acknowledged that the reliability and accuracy of in vivo clearance predictions from hepatocyte or microsomal data can be affected by the in vitro assay concentration of the drug. Concentrations around or above the K_M_ typically result in saturation of enzyme active sites and thus in enzyme kinetics that do not reflect the in vivo situation. In the field of radiotracer development, the discrepancy between standard assay concentrations (usually in the lower micromolar range) and in vivo radioligand concentrations (subnanomolar range) is particularly pronounced, which leads to further uncertainty regarding in vitro–in vivo extrapolation. In addition, the in vivo pharmacokinetics of tracer amounts of substance can deviate substantially from that of macro doses due to the existence of saturable enzyme and transporter systems as well as high affinity, low capacity binding sites [20,21]. Although PK dose-linearity has been successfully demonstrated for various pharmaceutical compounds in microdosing studies [22,23,24], the extremely high target affinities (usually nanomolar K_d_) exhibited by radiotracers developed for molecular brain imaging could potentially lead to deviations in pharmacokinetics between tracer and macro doses as a result of the long retention of the substance in the brain compartment which in turn reduces its hepatic exposure.

The present study evaluates the quantitative prediction of in vivo clearance from microsomal stability data in the rat preclinical model. The examined xanthine A_1_AR ligands represent ideal model compounds for in vitro-in vivo extrapolation approaches. As small (MW < 400 Da), neutral compounds of medium lipophilicity (log P: 2.2–2.9), CBX, MCBX and CPFPX can be classified as Class 2 drugs according to the extended clearance classification system (ECCS), for which metabolism is the predominant clearance mechanism [25].

The results from in vivo PK studies showed that, following i.v. administration, the three radiotracers were rapidly distributed to extravascular tissues with volumes of distribution that resembled total body water (approximately 600–700 mL/kg in male rats [26,27]). This indicates that the compounds are mainly subjected to hepatic metabolism and that plasma clearance can thus be assumed to be equal to hepatic clearance. Although the detailed physiological description of radiotracer disposition in the body is beyond the scope of this study, the existence of three distinct kinetic phases suggests radiotracer distribution between three compartments. The xanthine-based radiotracers can be assumed to cross biological membranes readily, which is confirmed by their relatively high V_d_-values; therefore, radiotracer distribution between a central plasma compartment and two tissue compartments with individual transport and equilibration characteristics appears to be a reasonable explanatory hypothesis.

The aggregation of individual plasma data into a mean data set enabled a more precise and robust estimation of PK parameters from triexponantial fits, since the influence of inherent noise present in the data was substantially reduced. This became particularly evident when calculating V_d_ and t_1/2,term_, which are derived from only one microconstant (λ_3_). The estimation of these parameters from fits of individual plasma curves repeatedly resulted in values which did not fall within physiologically reasonable ranges. The comparison between CL-values derived from mean curves and individual curves (deviation < 4%) clearly indicates that data aggregation is a valid approach in the context of this study.

Using the substrate depletion approach [28] and physiologically mechanistic scaling, in vivo clearance was predicted from in vitro stability data. The correlation between predicted and observed clearance was excellent for all three compounds, with only a slight underprediction of 1.3-fold. For comparison, a recent study which examined a large number of published datasets reporting in vitro CL_int_ and actual in vivo CL of classical pharmaceutical compounds reported an average underprediction of drug in vivo CL in RLM of 2.3-fold [29]. Additionally, when taking plasma protein binding into account, the rank order of in vivo metabolic stability could be accurately predicted from microsomal stability assays. In RLM, the rank order of metabolic stability (expressed by t_1/2_) was CBX > MCBX > CPFPX. When scaled to predicted CL_p_, the rank order changed to CBX < CPFPX < MCBX, with MCBX exhibiting higher clearance than CPFPX. This is in accordance with the actual in vivo observations, suggesting a substantial impact of plasma protein binding on the clearance of the model compounds. There is considerable controversy in literature on whether the extent of plasma protein binding correlates with clearance prediction accuracy. While several authors demonstrated a clear trend towards underprediction with highly bound drugs [30,31,32], others reported a lack of correlation between free fraction and prediction bias [29,33] or mixed effects depending on the physicochemical characteristics of the drug (acidic, basic or neutral) [28]. However, for the xanthine derivatives used in the present study, correction for plasma protein binding substantially improved the prediction of both clearance value and rank order. This can be explained by the specific physicochemical and pharmacological properties of these compounds. The combination of high plasma protein binding, moderate lipophilicity (which suggests medium membrane permeability) and relatively low intrinsic clearance (<Q) typically limits the hepatic extraction of a compound, which in turn affects its hepatic clearance [34,35,36].

The PK profiles of the novel cyclobutyl-substituted A_1_AR ligands differed distinctively from that of [^18^F]CPFPX. In the second and third phase of the curve (10-180 min p.i.), the plasma level of [^18^F]CBX was approximately twice as high as that of [^18^F]CPFPX, which corresponds to the considerably longer terminal half-life. In terms of imaging performance, this could potentially result in enhanced radiotracer delivery to the brain, since passive diffusion across the blood–brain barrier is driven by concentration. By contrast, [^18^F]MCBX showed a faster decline in plasma concentration in the first and second phase of the curve (0–40 min p.i.) than [^18^F]CPFPX. Although this could possibly lead to reduced brain exposure (depending on the extraction ratio of the radiotracer at the blood–brain barrier), reduced plasma radioactivity also diminishes background noise during the measurement, which improves the quality of the PET image. In view of these results, further evaluation studies should be conducted to assess the brain imaging performance of the novel A_1_AR radiotracers.

In conclusion, the present study underlines the value of in vitro metabolism models for radiotracer development. The data provide unequivocal evidence that accurate in vitro prediction of in vivo clearance is feasible despite concentration differences of more than three orders of magnitude between the in vitro and in vivo situation. This result encourages the implementation of in vitro stability studies as an integral part of the preclinical evaluation of novel PET radiotracers and suggests additional studies on the ability of human liver microsomes to a priori predict human radiotracer metabolism. Moreover, the novel cyclobutyl-substituted [^18^F]CPFPX analogs [^18^F]CBX and [^18^F]MCBX proved to be promising candidates for the development of A_1_AR radiotracers with enhanced imaging performance.

## 4. Materials and Methods

### 4.1. Compounds, Reagents and Solvents

CPFPX, CBX, MCBX, 8-cyclobutyl-3-(3-mesyloxypropyl)-7-pivaloyloxymethyl-1-propylxanthine (CBX precursor) and 3-(3-mesyloxypropyl)-8-(1-methylcyclobutyl)-7-pivaloyloxymethyl-1-propylxanthine (MCBX precursor) were synthesized and characterized in house as previously described [10,37]. 8-Cyclopentyl-3-(3-tosyloxypropyl)-7-pivaloyloxymethyl-1-propylxanthine (CPFPX precursor) was purchased from ABX GmbH (Radeberg, Germany). Reduced β-nicotinamide adenine dinucleotide 2′-phosphate (NADPH) was purchased from Roche Diagnostics (Mannheim, Germany). Magnesium chloride (MgCl_2_), 4-(2-hydroxyethyl)-1-piperazineethanesulfonic acid (HEPES), dimethyl sulfoxide (DMSO), acetic acid (HAc) and sodium hydroxide (NaOH) were purchased from Sigma-Aldrich (Steinheim, Germany). Reagent-grade methanol (MeOH), acetonitrile (ACN), ethyl acetate and hexane were obtained from Merck (Darmstadt, Germany). For preparation of eluents and buffers, aqua ad iniectabilia (water for injection) from B. Braun Melsungen (Melsungen, Germany) was used. Isoflurane for anesthesia was purchased from CP-Pharma (Burgdorf, Germany).

### 4.2. Animals

All animal experiments were conducted in accordance with the German Animal Welfare Act and approved by the governmental authorities (AZ: 84-02.04.2014.A496). Male Sprague Dawley rats (mean body weight at testing: 503 ± 44 g) were obtained from Charles River Laboratories (Sulzfeld, Germany). They were housed two to three per cage under standard conditions (12-h light/12-h dark cycle, 22 °C) with access to food and water ad libitum.

### 4.3. Radiochemistry

[^18^F]CPFPX was synthesized in house as described previously [10]. [^18^F]CBX and [^18^F]MCBX were synthesized analogous to [^18^F]CPFPX with minor adjustments of the chromatographic separation procedure (HPLC column: Kromasil 100-5 C18, 250 × 10 mm (AkzoNobel, Bohus, Sweden); eluent: MeOH/H_2_O/HAc 60:40:0.2 (*v/v/v*)). Radiochemical purity of all batches used for pharmacokinetic studies was >95%.

### 4.4. Microsomal Stability Assays

RLM (Sprague Dawley, male, 20 mg/mL protein concentration) were purchased from Thermo Fisher Scientific/Life Technologies (Darmstadt, Germany). Assay conditions (buffer, microsomal protein concentration, solvent) were evaluated and optimized in a preliminary study [37]. RLM (0.5 mg/mL microsomal protein) and substrate (8 µM CBX, MCBX or CPFPX, 1 mM stock solutions in DMSO) were preincubated for 5 min at 37 °C in HEPES buffer (100 mM, pH 7.4) containing MgCl_2_ (3.3 mM). Reactions were initiated by addition of preheated NADPH (1.3 mM). Final incubation volume was 1 mL. Aliquots (100 µL) were withdrawn at 0, 2, 5, 10, 15 and 20 min and quenched with an equal volume of methanol/acetonitrile (50:50, v/v, cooled to −20 °C). The samples were mixed on a vortex mixer (1 min, 21 °C) and centrifuged (20,000 rcf, 10 min, 21 °C) to sediment precipitated protein. The supernatants (aliquots of 50 µL) were analyzed on a Knauer smartline HPLC-UV/VIS system (Knauer, Berlin, Germany) equipped with a manual sample injector (Rheodyne type 7125), a 500 µL sample loop and a Kromasil 100-5-C18 (4.6 × 250 mm) analytical column (AkzoNobel, Bohus, Sweden). Chromatography was performed using a mobile phase composition of ACN/H_2_O/HAc 48:52:0.2 (*v/v/v*), and with a flow rate of 1 mL/min and a detection wavelength of 275 nm. Experiments were conducted in triplicate.

### 4.5. Data Analysis

Substrate depletion was calculated from the area ratios of the analyte peak, using the value at *t* = 0 min as 100%. Depletion data were fitted to the monoexponential decay model (Equation (1)) to derive in vitro *t*_1/2_.

(1) $$C\left(t\right)=C_{0}e^{-\frac{\ln2}{t_{1/2}}t}$$

where *C_0_* is the substrate concentration at time *t* = 0.

Intrinsic clearance was calculated from in vitro *t*_1/2_ using the equation [38]:

(2) $$CL_{int}=\frac{\ln2}{in\;vitro\;t_{1/2}\;\left(min\right)\times f_{mic}}\times \frac{ml\;incubation}{mg\;microsomal\;protein}\times \frac{mg\;microsomal\;protein}{g\;liver\;weight}\times \frac{g\;liver}{kg\;body\;weight}$$

where scaling factors of 60 mg of microsomal protein per gram of liver [39] and 40 g of liver tissue per kilogram of body weight [40] were applied.

The unbound fraction in microsomes was estimated using the following lipophilicity relationship algorithm [41]:

(3) $$f_{mic}=\frac{1}{1+P\times 10^{0.072\times \log P2+0.067\times \log P-1.126}}$$

where *P* is the microsomal protein concentration. The blood/plasma concentration ratio was assumed to be equal to 1 for the neutral xanthine compounds.

In vivo clearance in plasma was predicted using the well-stirred liver model [42,43]:

(4) $$CL_{p}=\frac{Q\times f_{p}\times CL_{int}}{Q+f_{p}\times CL_{int}}$$

where *f_p_* is the fraction unbound in plasma and *Q* is hepatic blood flow with a given value of 55 mL/min/kg for rat [40].

The individual prediction accuracy was assessed by calculation of fold error (ratio predicted/observed). AFE (Equation (5)) and RMSE (Equation (6)) were used as measures for overall bias and precision. Underprediction was also expressed as fold underprediction, which is the inverse of AFE.

(5) $$AFE=10^{\frac{1}{n}\sum log\frac{predicted}{observed}}$$

(6) $$RMSE=\sqrt{\frac{1}{n}\sum {\left(predicted-observed\right)}^{2}}$$

with *n*, number of predictions.

### 4.6. In Vivo Pharmacokinetics

Pharmacokinetic experiments on rats (*n* = 27) were conducted under isoflurane anesthesia (1.5–2% in oxygen) with continuous monitoring of physiological parameters. Polyethylene catheters (PE 50; filled with heparinized saline) were implanted into the femoral artery for arterial blood sampling and the lateral tail vein for radiotracer application. [^18^F]CPFPX, [^18^F]MCBX or [^18^F]CBX (21 ± 2.5 MBq, 0.39 ± 0.20 nmol) was formulated in physiological saline (1 mL total volume) and administered over 1 min using a syringe pump (model 44, Harvard Apparatus, Holliston, MA, USA). Arterial blood samples (ca. 200 μL) were collected at regular time intervals throughout the 180-min experiment. The total blood sampling volume was kept below 10% of the circulating blood volume of the animal. Plasma was separated by centrifugation (3,000 rcf, 3 min, 21 °C), weighed and measured in a γ-counter (ISOMED 2100, MED Nuklear-Medizintechnik Dresden GmbH, Dresden, Germany) to calculate plasma radioactivity concentration. Fractions of unchanged radiotracer (parent fraction) and radiolabeled metabolites in plasma were assessed by radio-thin layer chromatography (TLC) analysis. Aliquots (45 μL) of plasma were mixed with 3 volumes of methanol/acetonitrile (50:50, v/v, 4 °C), vortexed (1 min, 21 °C) and centrifuged (20,000 rcf, 5 min, 21 C) to sediment precipitated protein. Aliquots (5 μL) of the supernatants were spotted on a TLC plate (SIL G-25, 10 × 20 cm, Macherey-Nagel, Düren, Germany). The TLC plate was developed with ethyl acetate/hexane, 75:25 (v/v), dried and subsequently imaged for 50 min with an electronic autoradiography system (InstantImager, Canberra-Packard, Rüsselsheim, Germany).

### 4.7. Pharmacokinetic Analysis

PK analysis was performed on decay and metabolite-corrected plasma radioactivity data of 8 ([^18^F]CBX, [^18^F]CPFPX) or 9 ([^18^F]MCBX) individual animals. Data of 2 animals could not be used for PK analysis due to paravenous radiotracer injection. Since interindividual variations in plasma kinetics within the test groups were relatively small, individual plasma data were combined to mean data sets for analysis. Assuming a specific density of 1 g/mL for plasma, radioactivity concentration was calculated and plotted against time. For data visualization, plasma radioactivity concentration was normalized to body weight and amount of injected radioactivity, yielding SUV. PK parameters were derived from the radioactivity concentration-time data via nonlinear regression analysis applying a triexponential model:

(7) $$C_{p}\left(t\right)=A_{1}e^{-\lambda_{1}t}+A_{2}e^{-\lambda_{2}t}+A_{3}e^{-\lambda_{3}t}$$

where *C_p_* is the plasma radioactivity concentration, *t* is time, *A_1_*, *A_2_*, and *A_3_* represent the y-intercepts of the distribution/elimination phases of the plasma concentration-time curve and *λ_1_*, *λ_2_*, and *λ_3_* represent the first-order rate constants of the phases.

Plasma clearance, volume of distribution and terminal half-life were calculated from the model parameters *A* and *λ* according to the following equations [44]:

(8) $$CL_{p}=\frac{D}{\int_{0}^{\infty }C_{p}\left(t\right)dt}=\frac{D}{\sum_{i=1}^{n}\frac{A_{i}}{\lambda_{i}}}$$

(9) $$V_{d}=\frac{Cl_{p}}{\lambda_{3}}$$

(10) $$t_{1/2,term}=\frac{ln\;2}{\lambda_{3}}$$

where *D* is the injected radioactivity and *λ_3_* is the terminal rate constant.

To validate the results obtained from fitting mean data sets, *CL_p_* was also calculated from individual PK profiles.

### 4.8. Plasma Protein Binding

The binding of the radiotracer to plasma proteins was assessed via ultrafiltration, using Microcon-30 kDa centrifugal filter units (Merck Millipore, Darmstadt, Germany). Prior to radiotracer administration, blood plasma (200–300 µL) was sampled from the animal, spiked with 5–6 kBq of the radiotracer solution and incubated for 1 h at 37 °C. Subsequently, 100 µL of the spiked plasma was loaded onto the filter units which were then centrifuged for 20 min at 14,000 rcf and 37 °C. Radioactivity in equal volumes (50 μL) of spiked plasma and filtrate was measured in a γ-counter to calculate free fractions. Significant differences between plasma free fractions were assessed by one-way analysis of variance (ANOVA) followed by a post-hoc Tukey test. The significance level was set to 0.05. Normal distribution of the data and homogeneity of variances were assumed.

## Figures and Tables

**Table 1:** Metabolic stability of the xanthine A_1_AR ligands in rat liver microsomes and predicted plasma clearance (CL_p_). Experiments were conducted in triplicate. f_mic_, unbound fraction in microsomes; CL_int_, intrinsic clearance; SD, standard deviation.

Compound	Log P	f_mic_ Calculated	In vitro t_1/2_ (SD) min	CL_int_ (SD) mL/min/kg	CL_p_ Predicted (SD) mL/min/kg
CBX	2.19	0.90	35.1 (1.7)	106.1 (5.4)	2.72 (0.13)
MCBX	2.82	0.82	19.8 (0.7)	204.1 (6.8)	7.76 (0.22)
CPFPX	2.93	0.80	14.0 (0.2)	295.0 (5.0)	4.70 (0.07)

**Table 2:** In vivo pharmacokinetic (PK) characteristics of the xanthine A_1_AR radiotracers following i.v. administration to rat and accuracy of in vitro–in vivo extrapolation. PK parameters were derived from mean plasma radioactivity-time curves generated from data of eight ([^18^F]CBX, [^18^F]CPFPX) or nine ([^18^F]MCBX) animals.

Compound	V_d_ ml/kg	t_1/2,term_ min	CL_p_ ml/min/kg	Fold Error CL	Fold Underprediction CL
[^18^F]CBX	356	76.5	3.22	0.84	1.2
[^18^F]MCBX	715	54.5	9.10	0.85	1.2
[^18^F]CPFPX	523	51.0	7.10	0.66	1.5

## References

[1] Pike V.W. (2009). PET radiotracers: crossing the blood-brain barrier and surviving metabolism. Trends Pharmacol. Sci..

[2] Huang Y., Hwang D.R., Narendran R., Sudo Y., Chatterjee R., Bae S.A., Mawlawi O., Kegeles L.S., Wilson A.A., Kung H.F. (2002). Comparative evaluation in nonhuman primates of five PET radiotracers for imaging the serotonin transporters: [^11^C]McN 5652, [^11^C]ADAM, [^11^C]DASB, [^11^C]DAPA, and [^11^C]AFM. J. Cereb. Blood Flow Metab..

[3] Laruelle M., Slifstein M., Huang Y. (2003). Relationships between radiotracer properties and image quality in molecular imaging of the brain with positron emission tomography. Mol. Imaging Biol..

[4] Brandon E.F., Raap C.D., Meijerman I., Beijnen J.H., Schellens J.H. (2003). An update on in vitro test methods in human hepatic drug biotransformation research: Pros and cons. Toxicol. Appl. Pharmacol..

[5] Jia L., Liu X. (2007). The conduct of drug metabolism studies considered good practice (II): In vitro experiments. Curr. Drug Metab..

[6] Lipscomb J.C., Poet T.S. (2008). In vitro measurements of metabolism for application in pharmacokinetic modeling. Pharmacol. Ther..

[7] Pelkonen O., Turpeinen M. (2007). In vitro-in vivo extrapolation of hepatic clearance: biological tools, scaling factors, model assumptions and correct concentrations. Xenobiotica.

[8] Zhang D., Luo G., Ding X., Lu C. (2012). Preclinical experimental models of drug metabolism and disposition in drug discovery and development. Acta Pharm. Sin. B.

[9] Müller C.E., Jacobson K.A. (2011). Xanthines as adenosine receptor antagonists. Handb. Exp. Pharmacol..

[10] Holschbach M.H., Olsson R.A., Bier D., Wutz W., Sihver W., Schüller M., Palm B., Coenen H.H. (2002). Synthesis and Evaluation of No-Carrier-Added 8-Cyclopentyl-3-(3-[^18^F]fluoropropyl)-1-propylxanthine ([^18^F]CPFPX): A Potent and Selective A_1_-Adenosine Receptor Antagonist for in Vivo Imaging. J. Med. Chem..

[11] Bauer A., Holschbach M.H., Cremer M., Weber S., Boy C., Shah N.J., Olsson R.A., Halling H., Coenen H.H., Zilles K. (2003). Evaluation of ^18^F-CPFPX, a novel adenosine A_1_ receptor ligand: In vitro autoradiography and high-resolution small animal PET. J. Nucl. Med..

[12] Bauer A., Holschbach M.H., Meyer P.T., Boy C., Herzog H., Olsson R.A., Coenen H.H., Zilles K. (2003). In vivo imaging of adenosine A_1_ receptors in the human brain with [^18^F]CPFPX and positron emission tomography. Neuroimage.

[13] Elmenhorst D., Elmenhorst E.M., Hennecke E., Kroll T., Matusch A., Aeschbach D., Bauer A. (2017). Recovery sleep after extended wakefulness restores elevated A_1_ adenosine receptor availability in the human brain. Proc. Natl. Acad. Sci. USA.

[14] Elmenhorst E.M., Elmenhorst D., Benderoth S., Kroll T., Bauer A., Aeschbach D. (2018). Cognitive impairments by alcohol and sleep deprivation indicate trait characteristics and a potential role for adenosine A_1_ receptors. Proc. Natl. Acad. Sci. USA.

[15] Kroll T., Elmenhorst D., Weisshaupt A., Beer S., Bauer A. (2014). Reproducibility of non-invasive A_1_ adenosine receptor quantification in the rat brain using [^18^F]CPFPX and positron emission tomography. Mol. Imaging Biol..

[16] Nabbi-Schroeter D., Elmenhorst D., Oskamp A., Laskowski S., Bauer A., Kroll T. (2018). Effects of Long-Term Caffeine Consumption on the Adenosine A_1_ Receptor in the Rat Brain: an In Vivo PET Study with [^18^F]CPFPX. Mol. Imaging Biol..

[17] Bier D., Holschbach M.H., Wutz W., Olsson R.A., Coenen H.H. (2006). Metabolism of the A_1_ adenosine receptor positron emission tomography ligand [^18^F]8-cyclopentyl-3-(3-fluoropropyl)-1-propylxanthine ([^18^F]CPFPX) in rodents and humans. Drug Metab. Dispos..

[18] Matusch A., Meyer P.T., Bier D., Holschbach M.H., Woitalla D., Elmenhorst D., Winz O.H., Zilles K., Bauer A. (2006). Metabolism of the A_1_ adenosine receptor PET ligand [^18^F]CPFPX by CYP1A2: implications for bolus/infusion PET studies. Nucl. Med. Biol..

[19] Kreft S., Bier D., Holschbach M.H., Schulze A., Coenen H.H. (2017). New potent A_1_ adenosine receptor radioligands for positron emission tomography. Nucl. Med. Biol..

[20] Klopf W., Worboys P. (2010). Scaling in vivo pharmacokinetics from in vitro metabolic stability data in drug discovery. Comb. Chem. High Throughput Screen..

[21] Bergström M., Grahnén A., Långström B. (2003). Positron emission tomography microdosing: A new concept with application in tracer and early clinical drug development. Eur. J. Clin. Pharmacol..

[22] Balani S.K., Nagaraja N.V., Qian M.G., Costa A.O., Daniels J.S., Yang H., Shimoga P.R., Wu J.T., Gan L.S., Lee F.W. (2006). Evaluation of microdosing to assess pharmacokinetic linearity in rats using liquid chromatography-tandem mass spectrometry. Drug. Metab. Dispos..

[23] Ni J., Ouyang H., Aiello M., Seto C., Borbridge L., Sakuma T., Ellis R., Welty D., Acheampong A. (2008). Microdosing assessment to evaluate pharmacokinetics and drug metabolism in rats using liquid chromatography-tandem mass spectrometry. Pharm. Res..

[24] Lappin G., Noveck R., Burt T. (2013). Microdosing and drug development: past, present and future. Expert Opin. Drug Metab. Toxicol..

[25] Varma M.V., Steyn S.J., Allerton C., El-Kattan A.F. (2015). Predicting Clearance Mechanism in Drug Discovery: Extended Clearance Classification System (ECCS). Pharm. Res..

[26] Ward L.C., Battersby K.J. (2009). Assessment of body composition of rats by bioimpedance spectroscopy: Validation against dual-energy x-ray absorptiometry. Scand. J. Lab. Anim. Sci..

[27] Cornish B.H., Ward L.C., Thomas B.J. (1992). Measurement of Extracellular and Total Body Water of Rats Using Multiple Frequency Bioelectrical Impedance Analysis. Nutr. Res..

[28] Obach R.S. (1999). Prediction of human clearance of twenty-nine drugs from hepatic microsomal intrinsic clearance data: An examination of in vitro half-life approach and nonspecific binding to microsomes. Drug. Metab. Dispos..

[29] Wood F.L., Houston J.B., Hallifax D. (2017). Clearance Prediction Methodology Needs Fundamental Improvement: Trends Common to Rat and Human Hepatocytes/Microsomes and Implications for Experimental Methodology. Drug. Metab. Dispos..

[30] Bowman C.M., Benet L.Z. (2016). Hepatic Clearance Predictions from In Vitro-In Vivo Extrapolation and the Biopharmaceutics Drug Disposition Classification System. Drug. Metab. Dispos..

[31] Ring B.J., Chien J.Y., Adkison K.K., Jones H.M., Rowland M., Jones R.D., Yates J.W., Ku M.S., Gibson C.R., He H. (2011). PhRMA CPCDC initiative on predictive models of human pharmacokinetics, part 3: Comparative assessement of prediction methods of human clearance. J. Pharm. Sci..

[32] Ye M., Nagar S., Korzekwa K. (2016). A physiologically based pharmacokinetic model to predict the pharmacokinetics of highly protein-bound drugs and the impact of errors in plasma protein binding. Biopharm. Drug Dispos..

[33] Hallifax D., Houston J.B. (2012). Evaluation of hepatic clearance prediction using in vitro data: Emphasis on fraction unbound in plasma and drug ionisation using a database of 107 drugs. J. Pharm. Sci..

[34] Baker M., Parton T. (2007). Kinetic determinants of hepatic clearance: Plasma protein binding and hepatic uptake. Xenobiotica..

[35] Bohnert T., Gan L.S. (2013). Plasma protein binding: From discovery to development. J. Pharm. Sci..

[36] Smith D.A., Di L., Kerns E.H. (2010). The effect of plasma protein binding on in vivo efficacy: Misconceptions in drug discovery. Nat. Rev. Drug Discov..

[37] Schneider D., Bier D., Bauer A., Neumaier B., Holschbach M. (2019). Influence of incubation conditions on microsomal metabolism of xanthine-derived A_1_ adenosine receptor ligands. J. Pharmacol. Toxicol. Methods.

[38] Obach R.S., Baxter J.G., Liston T.E., Silber B.M., Jones B.C., MacIntyre F., Rance D.J., Wastall P. (1997). The prediction of human pharmacokinetic parameters from preclinical and in vitro metabolism data. J. Pharmacol. Exp. Ther..

[39] Carlile D.J., Zomorodi K., Houston J.B. (1997). Scaling factors to relate drug metabolic clearance in hepatic microsomes, isolated hepatocytes, and the intact liver: Studies with induced livers involving diazepam. Drug Metab. Dispos..

[40] Davies B., Morris T. (1993). Physiological parameters in laboratory animals and humans. Pharm. Res..

[41] Hallifax D., Houston J.B. (2006). Binding of drugs to hepatic microsomes: comment and assessment of current prediction methodology with recommendation for improvement. Drug Metab. Dispos..

[42] Rowland M., Benet L.Z., Graham G.G. (1973). Clearance concepts in pharmacokinetics. J. Pharmacokinet. Biopharm..

[43] Wilkinson G.R., Shand D.G. (1975). A physiological approach to hepatic drug clearance. Clin. Pharmacol. Ther..

[44] Yamaoka K., Nakagawa T., Uno T. (1978). Statistical moments in pharmacokinetics. J. Pharmacokinet. Biopharm..

